# Immune Reconstitution and Need for Booster Vaccinations Among Non‐Transplant Childhood Cancer Survivors: A Single‐Center Experience

**DOI:** 10.1002/cnr2.70326

**Published:** 2025-09-01

**Authors:** Esther Shin, Haesol Han, Kenneth J. Nobleza, Jonathan D. Crews, Araceli Elizalde, Nadia Cheek, Julie Voeller

**Affiliations:** ^1^ University of Incarnate Ward School of Medicine San Antonio Texas USA; ^2^ Baylor College of Medicine Houston Texas USA; ^3^ CHRISTUS Children's San Antonio Texas USA; ^4^ Allergy and Asthma Texas Health San Antonio Texas USA

**Keywords:** re‐immunization, survivorship, vaccinationimmune reconstitution

## Abstract

**Background:**

Waning immunity from childhood vaccines can be more profound in pediatric patients following chemo/immunotherapy. Moreover, childhood cancer survivors (CCS) are at significantly increased risk for life‐threatening infections. We implemented an institutional standard of practice (SOP) to assess immune reconstitution and provide recommendations for re‐vaccination for non‐transplant CCS.

**Methods:**

A lymphocyte mitogen proliferation panel was obtained at around 6 months off therapy to assess non‐specific lymphocyte proliferation to phytohemagglutinin (PHA), concanavalin A (Con A), and pokeweed mitogen (PWM). Serologic concentrations of immunoglobulin G (IgG) antibodies were measured for hepatitis B (hepB) virus, tetanus toxoid, and *Streptococcus pneumoniae* . Booster vaccines were recommended for seronegative patients. Catch‐up vaccines were recommended for CCS not previously up to date.

**Results:**

All evaluated patients were considered immune reconstituted at 6 months off therapy. Most patients (about 80%) were seronegative for hepB virus and *S. pneumoniae* , but nearly 100% retained immunity against tetanus. Most patients had positive seroconversion with a single booster vaccination with hepB (86%) and PPSV23 (76%). There were missed opportunities with each step of the institutional SOP due to provider omission with varying degrees depending on the provider.

**Conclusions:**

This study suggests that most CCS are ready for re‐immunization with inactivated vaccines by 6 months of therapy and underscores the need for boosters to optimize protection against vaccine‐preventable infections. Future studies are needed to inform consensus guidelines adapted to patient age, underlying cancer diagnosis, and treatment intensity.

**Abbreviations**
AAP: American Academy of Pediatrics
CCS: childhood sancer survivor
Con A: concanavalin A
DTaP: diphtheria‐tetanus‐pertussis vaccine
hepB: hepatitis B
HPV: human papillomavirus
IgG: immunoglobulin G
IPV: inactivated polio vaccine
IQR: interquartile range
MMR: measles, mumps, and rubella
PCV20: Prevnar 20
PHA: phytohemagglutinin
PPSV23: 23 pneumococcal serotypes
PWM: pokeweed mitogen
SOP: standard of practice

*S. pneumoniae*

:
*Streptococcus pneumoniae*

Tdap: tetanus‐diphtheria‐pertussis vaccine

## 1 Introduction

Childhood cancer survivors (CCS) are at increased risk for vaccine‐preventable infections as well as more severe and complicated infections leading to hospitalization or death [1, 2, 3, 4, 5]. Vaccination guidelines following hematopoietic stem cell transplant have been published [6], yet under‐immunization and increased hospitalizations for vaccine‐preventable infections are still prevalent [7]. Re‐vaccination of non‐transplant CCS is generally left to the provider's discretion, and without clear consensus guidelines, there is significant individual and institutional variability in practice [8]. There are possible strategies for re‐vaccination of non‐transplant CCS, with evidence of a protective response following boosters, but these studies have mostly been limited to survivors of leukemia [9, 10, 11, 12, 13, 14, 15].

Pediatric cancer patients have immune dysfunction due to their primary disease, such as in lymphoid malignancies, and exposure to antineoplastic therapies [1, 8]. Most patients recover immune function by 6 months after therapy; however, some remain deficient for years [1, 16]. Infectious complications, therefore, remain a significant cause of morbidity and mortality for pediatric cancer patients both on and off therapy.

Loss of humoral immunity against vaccine‐preventable diseases following cancer treatment has been well documented among CCS [17, 18, 19, 20, 21, 22, 23, 24]. Moreover, some patients are diagnosed with cancer prior to completing their primary vaccination series. Differences in loss of protective antibody titers, tempo of immune reconstitution, and response to re‐immunization following cancer treatment are known but less well defined [17, 21, 25, 26]. These differences can depend on the patient's age, underlying cancer diagnosis, type and intensity of treatment (particularly with the advent of cellular and immunotherapies), and type of vaccine.

Vaccinations are a highly effective public health strategy to protect individuals and communities from life‐threatening infections. Moreover, increased adherence to human papillomavirus (HPV) and hepatitis B virus (hepB) vaccination recommendations can also decrease morbidity and mortality from cancers associated with these viruses, for which CCS are at increased risk [3, 27].

To ensure optimal protection against vaccine‐preventable infections among non‐transplant CCS, we developed a unique institutional standard of practice (SOP) guideline for off‐therapy evaluation of immune reconstitution and need for re‐vaccination. In the absence of a gold standard, we opted to use a lymphocyte mitogen proliferation panel to comprehensively assess immune reconstitution. Without prior knowledge of baseline retention of immunity following chemotherapy, we optimally selected vaccine titers to assess the need for booster vaccination. Here we present our data on immune reconstitution, baseline seroprotection, and response to booster vaccination, as well as provider compliance with the institutional SOP. These data support the need for booster vaccination following chemotherapy for non‐transplant CCS and the need for future studies to establish clear consensus guidelines.

## 2 Methods

### 2.1 Patient Population

**FIGURE 1:**
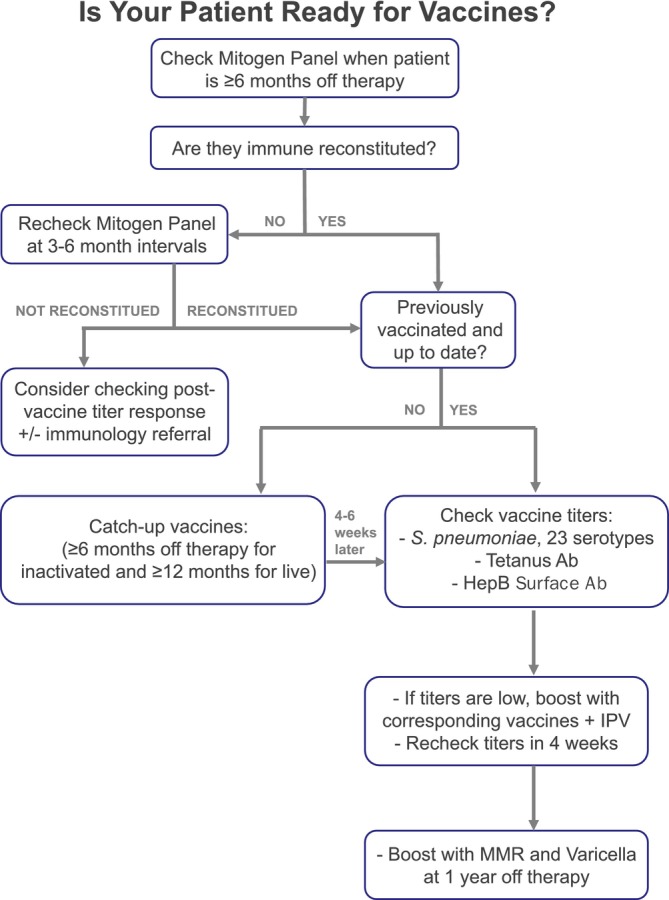
Institutional SOP. Flow diagram of our institutional standard of practice (SOP) for off‐therapy evaluation of immune reconstitution and baseline seroprotection and recommendations for re‐vaccination among non‐transplant childhood cancer survivors.

CHRISTUS Children's is the only free‐standing academic children's hospital in San Antonio, Texas. Between 60 and 80 new cancer cases are diagnosed yearly and treated per Children's Oncology Group protocols. An institutional SOP was established in 2019 as part of a quality improvement initiative to guide oncology providers in off‐therapy evaluation of immune reconstitution and need for re‐vaccination among non‐transplant CCS (Figure 1). Patients who completed their cancer treatment between July 2019 and October 2022 were eligible for this study. Patients were excluded from analysis if they had a diagnosis of relapsed cancer or immunodeficiency disorder, or if they declined vaccines. This study was approved by the CHRISTUS Health Institutional Review Board.

### 2.2 Assessment of Immune Reconstitution

A lymphocyte mitogen proliferation panel was obtained at around 6 months off therapy to assess non‐specific lymphocyte proliferation to phytohemagglutinin (PHA), concanavalin A (Con A), and pokeweed mitogen (PWM). Patients with at least a low response compared to a normal healthy control were considered to be immune reconstituted in this study [19, 28, 29, 30].

### 2.3 Assessment of Vaccination Coverage and Seroprotection

Vaccination status prior to the patient's cancer diagnosis was assessed based on parents' report of compliance with standard childhood vaccination guidelines per the American Academy of Pediatrics (AAP) and vaccination record when available. Antibody titers were not obtained prior to the initiation of chemotherapy.

If a patient had completed their age‐appropriate primary vaccination series prior to their cancer diagnosis, then serologic concentrations of IgG antibodies were measured at around 6 months off therapy for the following antigens: hepB, tetanus toxoid, and *Streptococcus pneumoniae* serotypes (PPSV23) 1, 2, 3, 4, 5, 6B, 7F, 8, 9N, 9V, 10A, 11A, 12F, 14, 15B, 17F, 18C, 19A, 19F, 20, 22F, 23F, and 33F. HepB antibody concentration was measured using the Architect instrument (Abbott, USA), while tetanus and *S. pneumoniae* antibody concentrations were performed by ARUP Laboratories (Salt Lake City, UT). Seroprotection against hepB and tetanus was defined as antibody concentrations ≥ 12 mIU/mL (per the manufacturer's package insert) and ≥ 0.1 IU/mL, respectively [31, 32]. In this study, long‐term seroprotection against *S. pneumoniae* was defined as an antibody concentration > 1.0 ug/mL for at least 50% of the 23 pneumococcal serotypes (or at least 12 serotypes) [33, 34].

If a patient had not yet completed their childhood vaccination series, completion of their primary series was recommended. Seroprotection against hepB, tetanus, and *S. pneumoniae* was then assessed as detailed above between 4 and 6 weeks after the final vaccine dose.

### 2.4 Recommendations for Booster Vaccines

Annual boosters for the influenza and COVID viruses were recommended regardless of immune reconstitution or seroprotection status. Patients with inadequate seroprotection against hepB, tetanus, or *S. pneumoniae* were recommended to receive an age‐appropriate booster dose of the corresponding vaccine: hepB (Engerix‐B, GlaxoSmithKline; or Recombivax HB, Merck), diphtheria‐tetanus‐pertussis vaccine with DTaP (Daptacel, Sanofi) or Tdap (Boostrix, GlaxoSmithKline; or Adacel, Sanofi), or PPSV23 vaccine (Pneumovax 23, Merck) [15]. Booster vaccination with PPSV23 was recommended over PCV13 due to its broader serotype coverage. Antibody levels were rechecked between 4 and 6 weeks after revaccination. Patients who did not attain positive serology were given a second booster dose of the corresponding vaccine, and serologic titers were again rechecked. Patients with a persistently inadequate response to vaccination were referred to immunology for further workup and management.

If any boosters of hepB, tetanus, or PPSV23 were recommended, an additional booster dose of inactivated polio vaccine (IPV, Sanofi; or Pediarix, GlaxoSmithKline if DTaP or hepatitis B vaccine also indicated) was recommended at ≥ 6 months off therapy as well as measles, mumps, and rubella (MMR) vaccine (MMR2, Merck) and varicella vaccine (Varivax, Merck) at ≥ 12 months off therapy [15].

### 2.5 Statistical Analysis

Descriptive data are reported as frequencies and proportions for categorical variables and as median (interquartile range, IQR) for continuous variables. The Fisher exact test was used to compare differences in baseline immunity and normalization by mitogen assay results. All analyses were performed on R v.4.3.1 (R Core Team, Vienna, Austria). A *p* value of < 0.05 was considered statistically significant for all statistical tests.

## 3 Results

### 3.1 Patient Characteristics

Of the 70 pediatric cancer patients who completed their treatment at CHRISTUS Children's Hospital from July 2019 to October 2022, 64 patients were included in this study (Table 1). Patients who had a known pre‐existing immunodeficiency syndrome (*n* = 1), refused vaccines (*n* = 3), or relapsed (*n* = 2) were excluded. A wide variety of cancer diagnoses were included, with the most common being leukemia (53%). The median age at cancer diagnosis was 5 years and 2 months. Forty‐three (67%) patients were up‐to‐date on vaccines at the time of cancer diagnosis, while 21 (33%) were not.

**TABLE 1:** Patient characteristics.

	Total	Leukemia	Lymphoma	Sarcoma	Other
	(*N* = 64)	(*n* = 34)	(*n* = 10)	(*n* = 4)	(*n* = 16)
Age at diagnosis (years)	5.2	4.9	14	7.9	4.7
Median (IQR)	(2.9, 13.6)	(3, 9.4)	(11.5, 16.1)	(1.1, 19.2)	(2.1, 11.0)
Primary vaccine series complete					
No	21 (32.8)	11 (32.4)	—	2 (50.0)	8 (50.0)
Yes	43 (67.2)	23 (67.6)	10 (100.0)	2 (50.0)	8 (50.0)
Low response on mitogen panel	18/57 (31.6)	11/31 (35.5)	4/9 (44.4)	1/4 (25.0)	2/13 (15.4)
Baseline HepB immunity					
Positive	10 (15.6)	3 (8.8)	—	1 (25.0)	6 (37.5)
Negative	36 (56.3)	23 (67.6)	6 (60.0)	2 (50.0)	5 (31.3)
Not done	18 (28.1)	8 (23.5)	4 (40.0)	1 (25.0)	5 (31.3)
Baseline tetanus immunity					
Positive	45 (70.3)	26 (76.5)	6 (60.0)	3 (75.0)	10 (62.5)
Negative	1 (1.6)	1 (2.9)	—	—	—
Not done	18 (28.1)	7 (20.6)	4 (40.0)	1 (25.0)	6 (37.5)
Baseline pneumococcal immunity					
Positive	10 (15.6)	3 (8.8)	3 (30.0)	—	4 (25.0)
Negative	37 (67.8)	24 (70.6)	3 (30.0)	3 (75.0)	7 (43.8)
Not done	17 (26.6)	7 (20.6)	4 (40.0)	1 (25.0)	5 (31.3)

*Note:* Values are in *n* (%) unless otherwise specified.

Abbreviations: HepB, hepatitis B; IQR, interquartile range.

### 3.2 Immune Reconstitution and Baseline Seroprotection

Of the 64 eligible and evaluable patients, 57 (89%) had a mitogen panel at around 6 months off therapy, and all were considered immune reconstituted. Seven patients did not have a mitogen panel administered due to loss to follow‐up (*n* = 1), patient refusal (*n* = 1), or the test not being ordered by the provider (*n* = 5).

Providers did not order hepB or tetanus titers for 18 (28%) patients, nor *S. pneumoniae* titers for 17 (27%) patients. Of the patients who were tested, 36/46 (78%) were seronegative for hepB, 1/46 (2%) was seronegative for tetanus, and 37/47 (79%) were seronegative for *S. pneumoniae* .

### 3.3 Booster Vaccination and Rates of Seroconversion

**FIGURE 2:**
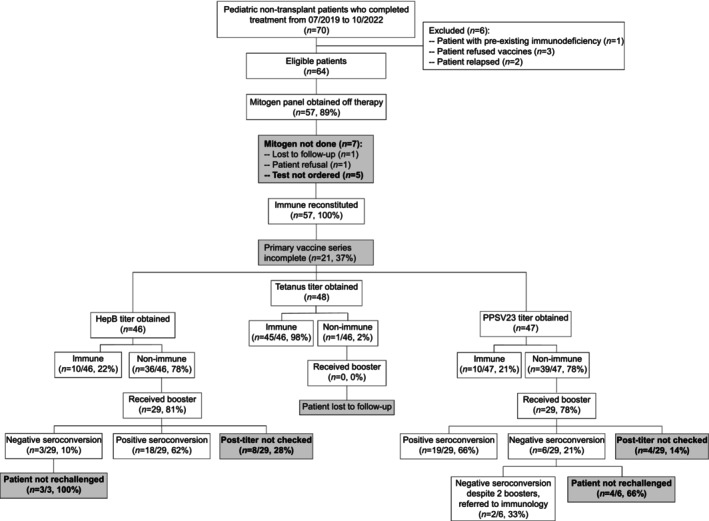
CONSORT diagram. CONSORT diagram of 64 eligible patients and rates of immune reconstitution, baseline seroprotection against hepB, tetanus, and *S. pneumoniae* , and response to booster vaccination. Grayed boxes with bolded text represent missed opportunities due to provider omission of the recommended step in the institutional SOP.

Of the 36 patients who were seronegative for hepB, 29 (81%) received a hepB booster (Figure 2). Most of the evaluated patients (18/21 or 86%) achieved adequate immunity following a single hepB booster. Two patients required more than one booster, and three patients did not have an adequate response after one booster but were not rechallenged or retested. Of the 29 patients who received a hepB booster, 8 (28%) patients did not have a titer rechecked following the booster.

The one patient who was seronegative for tetanus was lost to follow‐up.

Of the 37 patients who were seronegative for *S. pneumoniae* , 29 (78%) received a PPSV23 booster. Most of the evaluated patients (19/25 or 76%) achieved adequate immunity following a single PPSV23 booster. Four patients did not have an adequate response after one booster but were not rechallenged or retested. Two patients did not have an adequate response after two boosters and were referred to immunology. Of the 29 patients who received a PPSV23 booster, 4 (14%) did not have a titer rechecked following the booster.

There was a trend for patients with a low response on the mitogen panel to have inadequate baseline immunity to hepB (*p* = 0.07, Table 2). A low response on the mitogen panel did not affect patients' response to booster vaccinations with hepB (*p* = 0.43) nor PPSV23 (*p* = 0.28).

**TABLE 2:** Mitogen assay by baseline titer results.

Mitogen Panel	Baseline HepB immunity			Response to HepB booster		
	Immune, *n* = 10	Non‐immune, *n* = 36	*p*	Adequate, *n* = 18	Inadequate, *n* = 3	*p*
Normal	6 (66.7)	11 (31.4)	0.07	6 (35.3)	—	0.43
Low‐normal	—	12 (34.3)		6 (35.3)	2 (66.7)	
Low	3 (33.3)	12 (34.3)		5 (29.4)	1 (33.3)	

Mitogen Panel	Baseline pneumococcal immunity			Response to pneumococcal booster		
	Immune, *n* = 10	Non‐immune, *n* = 37	*p*	Adequate, *n* = 19	Inadequate, *n* = 6	*p*
Normal	5 (50.0)	13 (37.1)	0.75	9 (52.9)	1 (16.7)	0.28
Low‐normal	2 (20.0)	10 (28.6)		4 (23.5)	3 (50.0)	
Low	3 (30.0)	12 (34.3)		4 (23.5)	2 (33.3)	

*Note:* Values in *n* (%).

### 3.4 Provider Compliance With Institutional SOP

There were missed opportunities with each step of the institutional SOP due to provider omission with varying degrees depending on the provider. Of the 64 eligible and evaluable patients, 7 patients (11%) did not have a mitogen panel drawn at 6 months off therapy. Of the total 192 baseline vaccine titers recommended by the SOP (i.e., 3 baseline titers for each eligible and evaluable patient), 53 titers (28%) were missed. Of the 74 booster vaccinations recommended based on insufficient titers, 15 (20%) were missed. Of the 58 booster vaccines given, 19 (33%) post‐vaccination titers were not obtained to ensure adequate response.

## 4 Discussion

Timing of immune reconstitution and recommendations for re‐vaccination following chemo/immunotherapy for non‐transplant CCS are not well described in the literature. Current long‐term follow‐up guidelines recommend catch‐up vaccinations for any missed vaccines starting at 6 months off therapy, but the management of potential loss of vaccine‐related immunity following chemotherapy is left to shared decision‐making [35]. Without clear consensus guidelines, there is significant individual and institutional variability in practice [8]. Using a multidisciplinary collaborative approach including pediatric oncologists, immunologists, and infectious disease specialists, we developed an SOP (Figure 1) not only to standardize institutional practice but also to provide insight on immune reconstitution and baseline immunity among our patient population. In summary, we found that our patients were immune reconstituted by about 6 months off therapy, but most were not optimally protected against vaccine‐preventable diseases. This underscores the importance of booster vaccinations for non‐transplant CCS in the off‐therapy surveillance period. Of note, prior to this SOP, resuming the childhood vaccine schedule per AAP guidelines was recommended; booster vaccinations were not routinely recommended for non‐transplant CCS.

Effective immunizations must induce both the humoral and cell‐mediated arms of the adaptive system to efficiently produce effector and memory cells [36]. This complex process relies on an adequate number of functioning lymphocytes, which can take 12 or more months to recover following chemotherapy [19, 25]. Many methods have been used previously as a surrogate of immune reconstitution following chemotherapy, including absolute lymphocyte count, lymphocyte subset count, and immunoglobulin levels; however, these techniques represent a limited assessment of immune response [8, 12].

A mitogen panel assesses cellular immune response to PHA, Con A, and PWM, which are various plant‐derived mitogens that induce B and T lymphocyte activation [28]. We opted to check a mitogen panel at 6 months off therapy as the optimal single test to assess immune reconstitution, in the absence of a gold standard. Possible results include an absent, low, low‐normal, or normal response to these mitogens when compared to a healthy control. While the response to mitogens has been an effective measure to predict effective immune response to vaccinations [19], this test is sensitive to collection volume, shipping and handling, time elapsed since blood collection, and a variety of biological factors. Moreover, there is a practical challenge of comparing pediatric responses to adult healthy controls and reference values.

In this study, we considered a patient to be immune reconstituted if any response (i.e., at least low response) to mitogens was detected. All evaluated patients were immune reconstituted at 6 months off‐therapy, which is aligned with the general practice among most pediatric oncologists to resume catch‐up vaccinations with inactivated vaccines around this time. About a third of patients had a low response to mitogens, but this did not significantly impact patients' baseline immunity to hepB or *S. pneumoniae* nor patients' response to booster vaccination.

Various immunization strategies have been suggested post‐chemotherapy for non‐transplant CCS [8, 9, 10, 12]. In this study, we opted to obtain baseline vaccine titers for hepB, tetanus, and *S. pneumoniae* at around 6 months off therapy to investigate the immune status of our patient population. These titers were specifically chosen as these are well‐defined correlates of immunity, testing is readily available, and results are easy to interpret. Most of our patients were seronegative for hepB and *S. pneumoniae* , supporting our prediction that most of our CCS were not optimally protected against vaccine‐preventable infections. Of the patients evaluated, the majority of patients had positive seroconversion following a single booster of hepB (86%) and PPSV23 (76%).

Conversely, nearly all evaluated patients (98%) were seroprotected against tetanus, at rates higher than previously reported (46%–86%) [10, 12, 18, 37], except for one study which also found 100% protective anti‐tetanus levels post chemotherapy [26]. It is possible that the relatively longer half‐life of tetanus antibody (~10 years) and five‐dose vaccine schedule, with the most recent dose potentially given within a year prior to the cancer diagnosis, contributed to higher retention rates of tetanus immunity. Regardless, tetanus boosters are recommended even for immune competent persons because the bacteria is ubiquitous in the environment and highly infectious, and waning immunity over time is expected. Moreover, one study found the lowest retention of seroprotection against pertussis following chemotherapy [9], implicating the potential benefit of a single booster with DTaP or Tdap to provide additional protection against tetanus, diphtheria, and pertussis.

We opted to recommend booster vaccinations against polio, MMR, and varicella if any of the three measured titers (against hepB, tetanus, and *S. pneumoniae* ) were found to be inadequate, assuming the benefits of optimizing protection against these life‐threatening infections—in the setting of presumed waning seroprotection and increased endemic outbreaks—outweighed the costs and practical difficulty of obtaining serologic titers. Similarly, response titers were not obtained following booster vaccination with IPV, MMR, and varicella as prior studies have shown adequate response rates following a single booster dose [9, 10, 11].

There were missed opportunities with each step of the institutional SOP due to provider omission with varying degrees depending on the provider. This represents a difficulty in creating change, even amongst a relatively smaller group of five primary oncologists, without clear evidence‐based guidelines from international consortiums. There are also potential missed opportunities when patients reschedule or are lost to follow‐up.

Based on these data, we have opted to simplify our SOP by omitting potentially unnecessary and cost‐ineffective steps, such as obtaining a baseline mitogen panel and baseline immune titers [38]. We now recommend boosters for all routine vaccines for those who have completed their primary series (including the newer Prevnar 20 or PCV20 vaccine—which was not previously available during the time period of this study—due to longer‐lasting immunity as a conjugate vaccine while also providing broad serotype coverage). We subsequently obtain response titers for hepB and PPSV23. Of 27 patients in off‐therapy follow‐up since implementing this updated SOP in June 2024, 25 patients (93%) have received booster vaccinations and 22 patients (81%) have had post‐vaccine titers checked, an improvement from about 80% and 67%, respectively. In addition, we recommend the three‐dose HPV series regardless of prior vaccination status for all CCS given the higher risk for subsequent malignancies caused by HPV compared to the general population [35].

We did not measure antibody titers at the time of cancer diagnosis nor repeat titers to measure the persistence of immunity. We also did not formally perform a cost‐analysis comparing universal revaccination versus serologic testing and selective revaccination. In addition, it is difficult to account for the heterogeneity of oncologic diagnoses and immunosuppressive therapies.

In conclusion, this study suggests that most CCS can mount an appropriate immune response to vaccination by 6 months of therapy and underscores the need for boosters to optimize protection against vaccine‐preventable infections. Future studies are needed to create consensus guidelines adapted to patient age, diagnosis, and treatment intensity. Clearly defined recommendations will likely improve provider compliance and uniformity in our approach to optimize protection against vaccine‐preventable infections among CCS.

## Author Contributions

**Esther Shin:** data curation (equal), writing – original draft (equal), writing – review and editing (equal). **Haesol Han:** data curation (equal), writing – original draft (equal), writing – review and editing (equal). **Kenneth J. Nobleza:** data curation (equal), formal analysis (lead), writing – original draft (equal), writing – review and editing (equal). **Jonathan D. Crews:** conceptualization (equal), methodology (equal), writing – original draft (equal), writing – review and editing (equal). **Araceli Elizalde:** conceptualization (equal), methodology (equal), writing – original draft (equal), writing – review and editing (equal). **Nadia Cheek:** conceptualization (equal), data curation (equal), investigation (equal), methodology (equal), project administration (equal), supervision (equal), writing – original draft (equal), writing – review and editing (equal). **Julie Voeller:** conceptualization (equal), data curation (equal), formal analysis (equal), investigation (equal), methodology (equal), project administration (equal), supervision (equal), writing – original draft (lead), writing – review and editing (lead).

## Consent

This study was approved as a quality improvement project by the CHRISTUS Health Institutional Review Board.

## Conflicts of Interest

The authors declare no conflicts of interest.

## Data Availability

The data that support the findings of this study are available on request from the corresponding author. The data are not publicly available due to privacy or ethical restrictions.
